# Glances at the Hospitals

**Published:** 1900-10-06

**Authors:** 


### GLANCES AT THE HOSPITALS.

### HOSPITAL FOR WOMEN, WOLVERHAMPTON.

The income of this hospital for the year 1899 amounted to £991, but this sum included donations and bank interest; and the expenses came to £1,073, which shows that a deficiency of £81 existed on the year's working. The adverse balance now reaches £280. It is manifest that the committee do not ask unnecessarily for further subscriptions, and it seems almost incredible that these should not be forthcoming, as it is the only hospital of its kind in Staffordshire.

The tables give a clear description of the operations and the results of them. There were 203 operations, 83 being abdominal sections.

The hospital is not on the best possible site, but it will be removed to a more favourable one as soon as funds permit. Alderman Gibbons, M.P., has already given the land, and it is to be hoped that his lead will be followed as speedily and as liberally as possible. It is proposed, we understand, to build for not fewer than 25 beds, and this number is certainly not excessive when it is known that upwards of 4,000 patients were treated last year.

